# Preclinical evaluation of thermoreversible triamcinolone acetonide hydrogels for drug delivery to the inner ear

**DOI:** 10.1016/j.ijpharm.2014.05.057

**Published:** 2014-08-25

**Authors:** Elisabeth Engleder, Clemens Honeder, Julia Klobasa, Michael Wirth, Christoph Arnoldner, Franz Gabor

**Affiliations:** a Department of Pharmaceutical Technology and Biopharmaceutics, University of Vienna, Althanstraße 14, 1090 Vienna, Austria; b Department of Otorhinolaryngology, Medical University of Vienna, Währinger Gürtel 18, 1090 Vienna, Austria

**Keywords:** POX 407, Poloxamer 407, TAAc, Triamcinolone acetonide, DEX, Dexamethasone, POX 188, Poloxamer 188, ORO-test, Oscillation–rotation–oscillation test, Triamcinolone acetonide, Thermoreversible hydrogel, Poloxamer 407, Intratympanic administration, In-vitro release

## Abstract

Intratympanic glucocorticoid therapy aims to reduce the side effects associated with systemic long-time therapy of inner ear diseases or traumata after cochlear implantation. For that purpose, thermoreversible hydrogels being fluid at room temperature but solid at body temperature are known to be appropriate drug delivery systems. In this work, the two key parameters sol–gel transition time and temperature of Poloxamer 407 (POX 407) based hydrogels containing oto-compatible micronized triamcinolone acetonide (TAAc) were evaluated by rheological experiments varying the concentrations of the different compounds. A 20% POX 407 hydrogel in PBS containing 30% TAAc emerged as the most appropriate formulation. Oscillation–rotation–oscillation studies at two temperature levels were found to be an useful in-vitro test system for the hydrogel which revealed sufficient storage stability at 4 °C, injectability of the sol, solidification within 20 s at body temperature and persistent stiffness indicating prolonged adhesion at the round window membrane. According to the in-vitro release studies using the Transwell™ system, absorption of the poor water soluble TAAc is partly due to the low amount of dissolved drug but predominantly due to micellar transport resulting in a cumulative release of 262.6 ± 13.4 μg TAAc within one week followed by a sustained release of 193.1 ± 8.3 μg TAAc within the next three weeks. Thus, the formation of POX 407 micelles is the basis not only for gel formation but also absorptivity of TAAc. All in all, fine tuned rheological experiments and absorption studies emerged as useful tools for preclinical evaluation of intratympanally administered hydrogels.

## Graphical abstract

**Figure:**
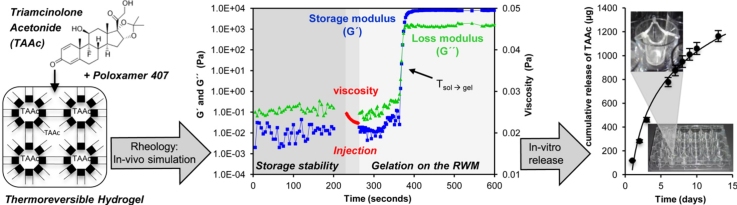

## 1 Introduction

As opposed to conventional systemic therapy, which suffers from pronounced side effects in case of long-term treatment, local glucocorticoid therapy of inner ear diseases or traumata after cochlear implantation is a new and patient-friendly approach (Bird et al., 2007). Unfortunately, unfavorable physiological conditions such as low blood flow in the cochlea as well as the blood–perilymph barrier limit therapeutic efficacy of drugs in cochlear fluids. In an effort to reach therapeutically relevant drug levels in the inner ear aqueous solutions of dexamethasone or betamethasone were administered intratympanally. These formulations, however, suffered from rapid drainage through the Eustachian tube and thus low drug levels in the inner ear due to short contact time with the round window membrane (Bird et al., 2007, 2011; Ye et al., 2007). To counteract the latter, semi-solid formulations were administered directly on the round window membrane and led to improved drug delivery into the inner ear due to prolonged contact with the membrane (Wang et al., 2011). According to Plontke et al. (2002), the amount of drug permeating into the cochlea depends mainly on the residence time of the drug on the round window membrane.

Out of these reasons, the thermoreversible characteristics of the oto-compatible (Piu et al., 2010; Wang et al., 2009), FDA-approved and autoclavable Poloxamer 407 (POX 407) (Dumortier et al., 2006; Guzman et al., 2006), being fluid at room temperature and semi-solid at 37 °C, seems to be most convenient for intratympanic administration. POX 407 is a triblock copolymer consisting of two hydrophilic polyethylene glycol end chains and a hydrophobic polypropylene glycol core block. Upon increasing the temperature, POX 407 molecules arrange to form micelles which subsequently aggregate to become a semi-solid hydrogel (Dumortier et al., 2006).

In general, the anti-inflammatory and immunosuppressive effects of glucocorticoids are best known, and they exert long-term effects on the tissue reaction against foreign objects like cochlear implants (Enticott et al., 2011). POX 407 gels containing dexamethasone (DEX) have been successfully used in several studies (Wang et al., 2011; Piu et al., 2010; Wang et al., 2009; Salt et al., 2011), and proved oto-compatibility with preservation of hearing thresholds. However, to the best of our knowledge there is a lack of studies about POX 407 gels containing triamcinolone acetonide (TAAc) for intratympanal administration although it is well established in clinical use, considered safe and preserves residual hearing together with a protective effect on hair cells (Ye et al., 2007; Guzman et al., 2006). The concentrations of TAAc applied in this study were related to the relative glucocorticoid potential of DEX as well as the release of DEX in therapeutically relevant levels as demonstrated by Piu et al. (2010) so that the efficacy of formulations containing 6% DEX and 30% TAAc might be comparable.

The aim of this work was to develop and characterize POX 407 based hydrogels with emphasis on the sol–gel transition temperature and sol–gel transition time upon varying the POX 407- and the TAAc-content. For that purpose, different rheological experiments as well as an adopted release model were applied. Additionally, the utility of additives such as POX 188, Miglyol, inorganic salts and ions (Dumortier et al., 2006; Müller Goymann and Luisana, 2011) to tune the sol–gel transition temperature is elucidated. All in all, this in-vitro study should offer a deeper insight into the structural characteristics as well as the release mechanisms of micelle-based hydrogels.

## 2 Materials and methods

### 2.1 Materials

Micronized triamcinolone acetonide (TAAc) was purchased from Fagron (Barsbüttel, Germany). Micronized dexamethasone (DEX) was obtained from Gatt-Koller (Absam, Austria). Pluronic^®^ F-127 Prill (Poloxamer 407, POX 407) was bought from BASF (Lampertheim, Germany). Pluronic^®^ F-68 (Poloxamer 188, POX 188) was acquired from Sigma–Aldrich (Vienna, Austria). 24-well-plates and filter inserts (ThinCert™ - 24 Well, Pore ø 0.4 μm, translucent, PET Membrane Ro Trac^®^) were from Greiner (Kremsmünster, Austria). All other chemicals were purchased from Sigma–Aldrich and were of analytical grade.

### 2.2 Methods

#### 2.2.1 Hydrogel preparation

The hydrogel was manufactured according to Schmolka (1972) by the “cold technique” using 10 mM phosphate buffered saline (PBS) pH 7.4 instead of water, and the POX 407 solution has been stored overnight at 4 °C prior use. Drug-loaded gels were prepared by homogeneously dispersing micronized particles of either TAAc or DEX (180 or 20 μm) in the fluid gel matrix by vortexing and storing at 4 °C overnight prior use.

#### 2.2.2 Sol–gel transition time

Thermosensitivity of the POX 407 hydrogel was determined by incubating 15–25% POX 407 solutions in PBS at 37 °C. In brief, 100 μl POX 407 solution at 4 °C was transferred into an Eppendorf cup and subsequently incubated at 37 °C. After 5, 10, and 20 min, the Eppendorf cups were inverted and solidification was assessed.

#### 2.2.3 Rheological characterization

The reversibility of the sol–gel transition was investigated by placing the cold formulation (4 °C) into an Eppendorf cup, incubating for 15 min at 37 °C to become rigid, and then storing the gel in a refrigerator to liquefy again. This procedure was repeated 10 times. The state of aggregation was assessed after inverting the cup.

The hydrogel's rheological characteristics were examined with a Modular Compact Rheometer MCR 302 equipped with a cone/plate system (CP50-1-SN27364; 50 mm/1° cone plate; Anton Paar, Graz, Austria). All measurements were done in a Peltier controlled hood, and the data were processed using the Rheoplus/32 V3.61 software. The storage modulus *G*′ and the loss modulus *G*″ were calculated from the complex shear modulus G^*^ as follows:

(1) $$G^{\prime }=G^{*}\text{cos}(\delta )$$

(2) $$G^{\prime }=G^{*}\text{sin}(\delta )$$

(3) $$\text{tan}(\delta )=\frac{G^{\prime \prime }}{G^{\prime }}$$

To define the linear viscoelastic region (LVR) shear strain amplitude sweeps were carried out increasing the deformation from 0.001 to 100% at an angular frequency of 3 rad s^−1^ (Grüning and Müller-Goymann, 2008).

Frequency sweep experiments were performed at a constant deformation of 0.1% (20% POX 407; w/v) or 0.01% (20% POX 407 containing 30% TAAc; w/v) and decreasing the angular frequency from 100 to 0.01 rad s^−1^.

Temperature-dependent characteristics of the preparations were elucidated in the range from 4 °C up to 42 °C at a heating rate of 1 °C min^−1^ in the oscillatory mode setting the angular frequency at 3 rad s^−1^ and the deformation at 0.1% without and 0.01% with TAAc, respectively.

Additionally, an oscillation–rotation–oscillation (ORO) test was accomplished at the same oscillation settings as above. In brief, during the first oscillatory mode the formulation was maintained for 200 s at 4 °C until the temperature increased up to 10 °C within 30 s. Subsequently, the rotation mode was performed at 10 °C for 30 s at the shear rate of 5000 s^−1^, which simulates the injection process (injection needles LUER G27: 0.4 × 42 mm) and was calculated according to Eq. (4):

(4) $$\gamma =\frac{4\times V}{\pi \times R^{3}\times t}$$

where *γ* is the calculated shear rate, *V* is the effluent volume, *R* is the radius, and *t* is the time.

Finally in the second oscillatory mode, the bottom plate was warmed up to 37 °C (0.2 °C s^−1^) within 135 s and maintained at 37 °C for 120 s, subsequently the temperature was increased up to 42 °C.

All experiments, performed with a sample volume of 600 μl and at a cone gap of 0.101 mm, were repeated at least 3 times.

#### 2.2.4 Release studies

In a preliminary attempt to establish a release model without a membrane barrier, 150 μl of a 20% POX 407 hydrogel colored by addition of 0.005% aqueous Evans Blue were transferred into an Eppendorf cup and incubated for 15 min at 37 °C. The solidified gel was overlain with 70 μl 1% aqueous 2-ethoxy-6,9-diaminoacridine lactate solution of 37 °C and further incubated at body temperature for 24 h. The images were acquired at defined times.

As a release model including a barrier, translucent filter inserts in 24-well plates were applied using artificial perilymph as an acceptor medium. The aqueous artificial perilymph was composed of 137 mM NaCl, 5 mM KCl, 2 mM CaCl_2_, 1 mM MgCl_2_ and 1 mM NaHCO_3_, but without 11 mM glucose to avoid bacterial contamination.

After pre-soaking the polyethylene terephthalate membrane (0.4 μm pore diameter) of the filter inserts in artificial perilymph for 30 min at room temperature 100 μl cold, liquid gel were applied on the filter, and the well-plate was incubated for 20 min at 37 °C. After solidification of the hydrogel, 300 μl artificial perilymph were transferred to the acceptor chamber so that the artificial perilymph was at the same level as the membrane of the filter insert.

At predetermined time points 200 μl aliquots were withdrawn from the acceptor compartment. Additionally, in an effort to collect the entire permeated drug and to dissolve precipitated corticoid, the filter insert was removed and the residual fluid in the lower chamber was mixed with 300 μl mobile phase. These two samples were pooled to yield finally 600 μl sample per time point to be assayed by HPLC. To continue the release study, the filter insert was transferred to a new well, and 300 μl fresh artificial perilymph were added.

The release studies were carried out at 37 °C under low-germ condition and repeated 3 times. Statistical analyses were carried out by using ANOVA.

#### 2.2.5 Quantitation of TAAc and DEX

The amount of released drug was determined by UV-detection at 240 nm following HPLC using an Agilent 1100 System (Agilent Technologies, Palo Alto, USA) equipped with a vacuum degasser, a quaternary pump, an autosampler, a column compartment thermostated at 25 °C, a diode array detector and the Agilent ChemStation software. 20 μl of the sample were separated in an Acclaim^®^ 120 C18 reversed-phase LC column (2.1 mm × 150 mm, 3 μm; Thermo Fisher Scientific, Vienna, Austria) at a flow rate of 0.5 ml min^−1^ using a mobile phase of 60 parts acetonitrile and 40 parts 2 mM aqueous ammonium acetate, pH 3.2 adjusted with formic acid (TAAc) or 40 parts bidistilled water (DEX), respectively (César et al., 2011). The retention time was 1.75 and 1.37 min for TAAc and DEX, respectively. The overall runtime was 5 min.

Calibration graphs using mobile phase containing 50% artificial perilymph with or without 1% human serum albumin (HSA) (Swan et al., 2009) as a diluent were prepared in the range of 1–100 μg ml^−1^ TAAc. The quality control samples were made in the same way with defined amounts of drug in the range of the calibration graph.

## 3 Results

### 3.1 Sol–gel transition of the POX 407 formulation

**Fig. 1:**
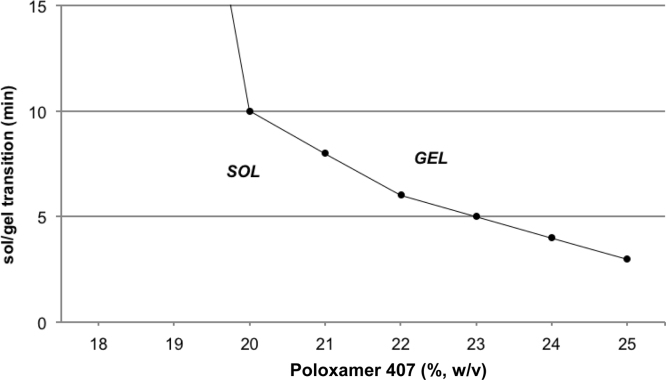
Sol–gel transition of the thermoreversible hydrogel assessed by the tube inversion method.

In general, the solidification time at 37 °C decreased with increasing POX 407 content, but at the same time the viscosity of the solutions at 4 °C increased making pipetting of the required small volume of 50 μl difficult (Fig. 1). Within the range of 15–25% solutions, 20% POX 407/PBS formulations possessed an optimal solidification time of about 10 min.

### 3.2 Amplitude sweep experiments

**Fig. 2:**
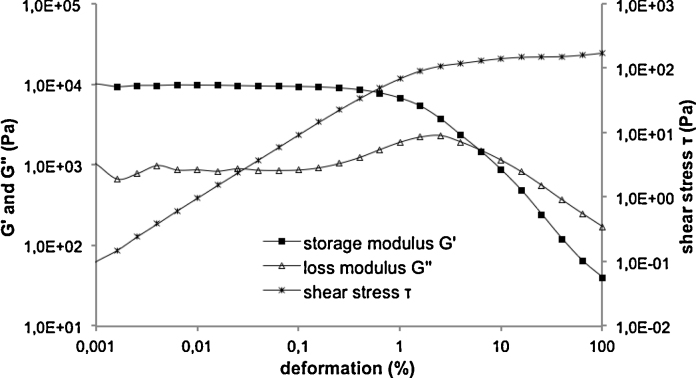
Amplitude sweep of 20% POX 407/PBS containing 30% TAAc at 37 °C showing the LVR characterized by the plateau and parallel behavior of *G*′ and *G*″.

The LVR of the 20% POX 407 preparations with or without TAAc was determined by measuring the storage modulus (*G*′) and the loss modulus (*G*″) as a function of a shear strain amplitude sweep at 37 °C. According to Mezger (2011), the viscoelastic state is the region where *G*′ exceeds *G*″ and the LVR is characterized by a plateau and parallel behavior of *G*′ and *G*″ (Fig. 2). The end of the LVR, the so-called yield point, is the point where *G*′ decreases indicating the beginning of alteration of the internal structure of the hydrogel. In case of 20% POX 407-hydrogels/PBS containing 30% TAAc, the LVR ends at 0.16% deformation whereas without the drug, the yield point was at 0.39%. Consequently, to retain the internal structure during the measurements, in all further experiments the deformation was set at 0.01% for hydrogels containing TAAc and at 0.1% for hydrogels without the drug, respectively.

### 3.3 Frequency sweep experiments

**Fig. 3:**
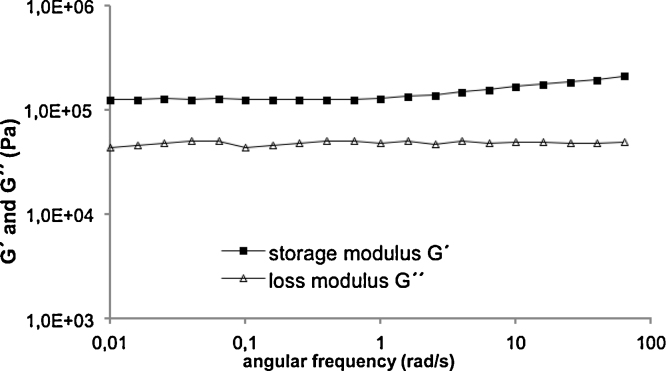
Frequency sweep of 20% POX 407/PBS containing 30% TAAc at 37 °C.

To simulate stability of the hydrogel after intratympanic administration frequency sweep experiments of 20% POX 407/PBS preparations without and with 30% TAAc were performed at 37 °C. As required for hydrogels, the storage modulus exceeded the loss modulus by about 74,400 Pa, and the course of the curve was nearly parallel to the *x*-axis at low frequency ranges from 0.01 rad s^−1^ up to 20 rad s^−1^ (Fig. 3). As a rule of thumb, storage moduli >10 Pa at 0.01 rad s^−1^ indicate stability of dispersions. Thus, the storage moduli of the hydrogel with and without TAAc amounting to 123,000 Pa and 4860 Pa additionally confirm preservation of the gel structure.

### 3.4 Temperature sweep experiments

The influence of sweeping the temperature on storage modulus and loss modulus was investigated for various POX 407-based hydrogels in order to determine the specific sol–gel transition temperature (*T*_sol_→*T*_gel_). At 4 °C all preparations were Newtonian liquids as indicated by loss moduli exceeding the storage moduli. Upon increasing the temperature, the *T*_sol_→*T*_gel_ is reached where the storage module is equal to the loss module and the fluid is converted into a plastic solid. Concurrently, the shear stress steeply increases. Further rise of the temperature inverts the initial ratio between loss and storage moduli to become finally independent from temperature. According to Table 1, the presence of salts decreases the *T*_sol_→*T*_gel_ by 3.4 °C, whereas the addition of 6–30% steroid has no effect. In accordance with the literature (Dumortier et al., 2006; Müller Goymann and Luisana, 2011), Miglyol as well as POX 188 increase the *T*_sol_→*T*_gel_ by 1.4 °C and 5.4 °C, respectively. Although the *T*_sol_→*T*_gel_ was not altered by the amount of steroid added, the stiffness of the hydrogel as indicated by the complex shear modulus is quite different as compared to the hydrogel without TAAc; this parameter is nearly constant for 6% TAAc but increases 2.3-fold in case of 30% steroid-content.

### 3.5 Oscillation–rotation–oscillation experiments

**Fig. 4:**
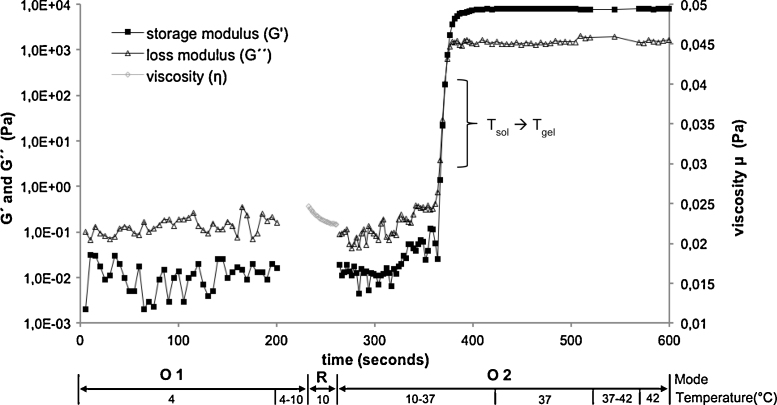
ORO test of a 20% POX 407/PBS hydrogel containing 30% TAAc to simulate the intratympanal application. The first oscillation mode (“O1”) indicates long-term stability at 4 °C, the rotation mode (“*R*”) exerts high shear forces occurring during injection at 10 °C, and the second oscillation mode (“O2”) represents the gelation process upon increasing the temperature and the stability of the hydrogel at the round window membrane.

To estimate in-vitro the influence of high shear forces on the thixotropic behavior during intratympanic injection ORO-studies were performed (Fig. 4). Concurrently, the influence of body temperature was assessed by adjusting the temperature levels. During the first oscillation mode within the LVR at 4 °C representing storage conditions, the preparations were fluid as indicated by 10-fold higher loss moduli as compared with the storage moduli. The shear forces in the course of squeezing the preparation through the injection needle were imitated by the subsequent rotation mode at 10 °C leading to a decrease in viscosity. Finally, the situation at the round window membrane is mimicked by the second oscillation mode at 37 °C. Due to the increased temperature, the preparations became semi-solid as indicated by inversion of the ratio between loss modulus and storage modulus in agreement with the results of the temperature sweep studies. Finally, after reaching the *T*_sol_→*T*_gel_ transition, the preparations became solid within 20 s, and the storage modulus exceeded the loss modulus about 10-fold. In comparison with the temperature sweep experiments, the *T*_sol_→*T*_gel_ transition now shifted from 25.1 °C to 28 °C (20% POX 407/PBS) or from 25 °C to 28.9 °C (20% POX 407/PBS with 30% TAAc) due to previous rotation. Prolonging the oscillation and increasing the temperature up to 42 °C did not alter the storage, and the loss modulus so that a stable consistency at the site of administration can be expected.

### 3.6 Release studies

As a prerequisite for release studies, the HPLC-method for quantitation of the steroids was adapted and validated. The calibration graphs revealed excellent linearity for TAAc in presence and absence of human serum albumin (*R*^2^ = 0.9994 and *R*^2^ = 0.9998) and DEX (*R*^2^ = 0.9997) in the concentration range of 1–100 μg ml^−1^. The addition of HSA had no significant influence (*p* > 0.05) on the TAAc quantification. The lower limit of detection and the lower limit of quantitation was 0.1 μg ml^−1^ and 1 μg ml^−1^ in case of TAAc, 0.05 μg ml^−1^ and 0.1 μg ml^−1^ in case of DEX. The inter-day precision for 15 days and the intra-day precision was 2.1% or 0.9% in case of TAAc and 1.9% or 1.8% in case of DEX.

In a preliminary assay, the applicability of the release model without barrier was assessed roughly by covering the blue dyed hydrogel with yellow release medium. Within 24 h, a green supernatant was observed indicating dissolution of the hydrogel. Consequently, a barrier representing the round window membrane is necessary to approach in-vivo conditions.

**Fig. 5:**
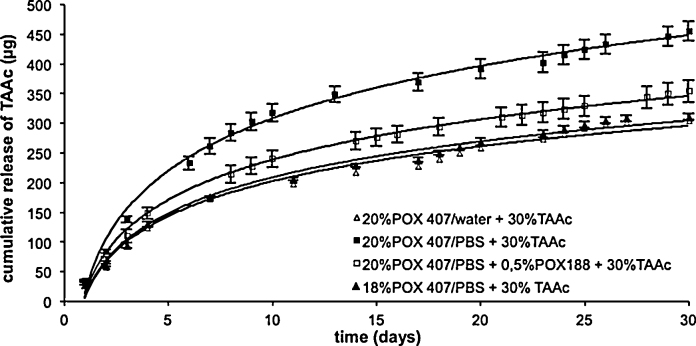
In-vitro release profiles of different POX 407 hydrogels containing 30% TAAc using the trans-well system with a polyethylene terephthalate membrane over a period of 30 days (mean ± SD < 10.9).

Using a membrane with 0.4 μm pore diameter and 30 μm thickness as a barrier, the cumulative release of TAAc from the individual POX 407 hydrogels after 30 days was in the range from 1.0% to 1.6% (Fig. 5). In detail, however, the total amount of drug released from 30% TAAc hydrogels was quite different: lowest release with no significant difference (p  > 0.05) was observed in case of 18% POX 407/PBS and 20% POX 407/water amounting to 311.3 ± 4.9 μg TAAc and 305.3 ± 8.9 μg TAAc, respectively. Incorporation of 0.5% POX 188 in 20% POX 407/PBS hydrogels increased the cumulative release to 355.0 ± 18.7 μg TAAc. Among all the formulations investigated, 20% POX 407/PBS hydrogels exhibited highest cumulative release of 455.7 ± 16.2 μg TAAc.

**Fig. 6:**
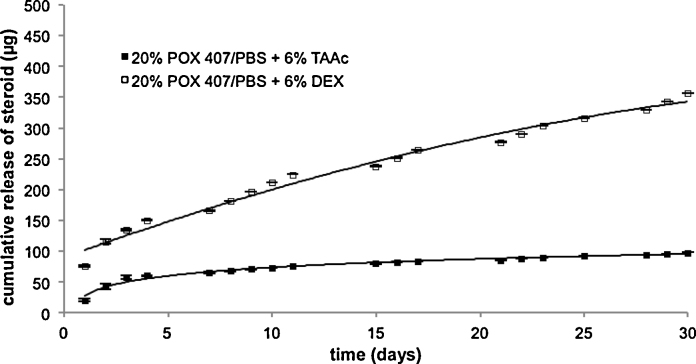
In-vitro release profile of 20% POX 407/PBS containing 6% DEX or 6% TAAc over a period of 30 days. (mean ± SD ≤ 4.40).

For comparison, the release profiles of 20% POX 407/PBS hydrogels loaded with either 6% TAAc or DEX were examined (Fig. 6). Applying the same conditions as above, the cumulative release of TAAc was 1.6 ± 0.1% corresponding to 98.0 ± 5.3 μg after 30 days as opposed to 5.9 ± 0.2% corresponding to 356.6 ± 9.4 μg drug in case of DEX. Besides, the saturation concentration of TAAc in AP at 37 °C is 12.1 ± 0.3 μg ml^−1^ or 139.89 ± 1.66 μg ml^−1^ in AP containing 5% POX 407. All release profiles follow the kinetic model of Higuchi (Higuchi, 1961) with a coefficient of determination >0.943 or >0.897 for the hydrogel containing 6% TAAc.

## 4 Discussion

The closely interacting key-parameters for applicability of in-situ forming thermoreversible drug delivery systems in the inner ear are the time and the temperature required for sol–gel transition. On the one hand, the time frame should be long enough to allow save intratympanic administration of the preparation, on the other hand duration of surgery should be as short as possible to reduce the length of anesthesia during cochlear implantation. The solidification time has to be fine tuned with the sol–gel transition temperature (*T*_sol_→*T*_gel_), as the solution should become semi-solid at body temperature as soon as possible to avoid loss by Eustachian drainage.

A simple tube inversion test as well as rheology revealed that a 20% POX 407/PBS solution gels within 10 min at 37 °C. As elucidated by temperature sweep experiments, the *T*_sol_→*T*_gel_ increased with decreasing POX 407 content, because gelation is promoted by higher availability of POX 407 micelles (Dumortier et al., 2006; Ricci et al., 2002). The addition of even high amounts of steroid did not alter the *T*_sol_→*T*_gel_, but provoked a tenfold lower deformation of the hydrogel. This increase in stiffness due to presence of drug microparticles (30% TAAc) in between the micelles is also displayed by a complex shear modulus exceeding that of the plain hydrogel 1.3 fold. In spite of the high solid content, the stability of the hydrogels was not adversely affected since the course of the storage moduli was still parallel to the *x*-axis at low frequencies.

Moreover, the presence of ions and inorganic salts being components of PBS decreased the *T*_sol_→*T*_gel_ by 3.4 °C in comparison to ion-free formulations. According to Ricci et al. (2002), the affinity of water to ions is higher than that to POX 407 so that the lack of water molecules in between the micelles facilitates gelation resulting in lower *T*_sol_→*T*_gel_. Concurrently, the complex shear modulus increased by a third, which points to increased stiffness as well as bioadhesiveness according to the literature (Dumortier et al., 2006; Ricci et al., 2002; Choi et al., 1999). Especially the latter effect is supposed to be beneficial since a long contact time at the round window membrane is desired.

A quite opposite effect on *T*_sol_→*T*_gel_ was observed upon addition of POX 188 or Miglyol. These substances increase the gelation temperature and have been reported to disturb the micellization process (Dumortier et al., 2006; Choi et al., 1999). In general, frequency sweep experiments confirmed long-term stability of all tested hydrogels. Altogether, the 20% POX 407/PBS hydrogel containing 30% TAAc emerged to be the most appropriate formulation.

The oscillation–rotation–oscillation experiments at temperature levels chosen according to the expected environment in the middle ear proved to be most useful for prediction of the practical applicability of the hydrogels. The rheological parameters during the first oscillation mode at 4 °C confirmed storage stability of the solutions, the rotation mode at 10 °C mimicking high shear forces proved injectability of the formulation, and finally the second oscillation mode at 37 °C simulated the situation at the round window membrane. Upon contact with the absorption barrier, the formulation undergoes gelation after a short delay of 19.5 s, and the inner structure of the gel is maintained as tan *δ* remains constant indicating long-term stiffness of the hydrogel and thus prolonged residence at the membrane.

The subsequent absorption of TAAc at the round window membrane was simulated applying a membrane model with a pore diameter smaller than that of the drug microparticles. Considering that the flow of the perilymph is about 2–4 nl min^−1^, and the estimated volume is 12–16 μl (Schaaf, 2012; Salt et al., 2006), 300 μl artificial perilymph were used as an acceptor medium. This volume is a compromise between assay practicability and physiology. Nevertheless, non-sink conditions prevail in-vitro and in-vivo as the saturation concentration was determined to be about 10 μg TAAc/ml artificial perilymph at 37 °C.

The release mechanism is supposed to be guided by the spatial distribution of TAAc in the gel matrix: (i) the dissolved proportion of TAAc accumulates in the hydrophobic core of the micelles. In case of a 20% POX 407/PBS hydrogel, up to 6% TAAc might be incorporated into micelles as the stiffness of the hydrogel as represented by the complex shear modulus was comparable to that without drug. (ii) The insoluble, micronized proportion of the steroid is distributed in between the POX 407 micelles. Consequently, the absorption of TAAc might be mainly due to micellar transport. This assumption is underlined by the fact that the cumulative release of TAAc from 20% POX 407/PBS hydrogels exceeds that from 18% hydrogels by 68% due to higher availability of POX 407 micelles. Additionally, after micellar transport across the membrane, the TAAc precipitated in the acceptor compartment, because the POX 407 concentration deceeds the critical micelle concentration, and the TAAc exceeds its saturation solubility. Moreover, the insoluble drug contributes to increased stiffness of the hydrogel as indicated by a 2.3-fold higher complex shear modulus of the 30% TAAc hydrogel.

At similar POX 407 concentrations, the cumulative release from a 6% and a 30% TAAc hydrogel was only slightly different amounting to 1.6 ± 0.1% and 1.5 ± 0.1%. However, the cumulative amount of permeated drug from 30% TAAc hydrogels was 4.6-fold higher. The presence of POX 188 reduced the cumulative release by about a fifth due to disturbance of micellization as already observed during rheological examinations.

Interestingly, at 6% drug loading, the cumulative release of DEX was 3.6-fold higher than that of TAAc. Thus, solubility is another parameter influencing release from Poloxamer hydrogels, as the aqueous solubility of DEX is reported to be 10–1000 times higher than that of TAAc (Block and Patel, 1973; Behl et al., 1976).

In general, the in-vitro release profiles indicate an initial burst within the first week releasing up to 262.6 ± 13.4 μg TAAc followed by a sustained release of 193.1 ± 8.3 μg TAAc within the next three weeks, which might also be beneficial for in-vivo administration.

## 5 Conclusion

All in all, oscillation–rotation–oscillation experiments to simulate storage stability, injection as well as solidification of the formulation at the site of administration combined with release studies using the Transwell™ system to mimic the in-vivo drug transport were found to be most useful tools to establish and optimize TAAc-loaded thermoreversible hydrogels for intratympanal administration.

## Figures and Tables

**Table 1:** Sol–gel transition temperatures of various POX 407 based hydrogels.

Gel type	Addition of	Sol–gel-transition temperature (°C, mean ± 0.75)	Δ °C cf/20% POX 407/water	Complex shear modulus at 37 °C
20% POX 407/water		28.0	0.0	8820
20% POX 407/PBS		24.6	−3.4	12,433
20% POX 407/PBS	30% TAAc	24.4	−3.6	29,100
20% POX 407/PBS	6% TAAc	24.8	−3.2	12,100
20% POX 407/PBS	6% DEX	24.5	−3.5	10,400
20% POX 407/PBS	1% Miglyol	26.0	−2.0	11,700
20% POX 407/PBS	0.5 POX 188	30.0	+2.0	9260
18% POX 407/PBS		27.4	−0.6	6150
